# An Ensemble Method with Hybrid Features to Identify Extracellular Matrix Proteins

**DOI:** 10.1371/journal.pone.0117804

**Published:** 2015-02-13

**Authors:** Runtao Yang, Chengjin Zhang, Rui Gao, Lina Zhang

**Affiliations:** 1 School of Control Science and Engineering, Shandong University, Jinan, China; 2 School of Mechanical, Electrical and Information Engineering, Shandong University at Weihai, China; University of Crete, GREECE

## Abstract

The extracellular matrix (ECM) is a dynamic composite of secreted proteins that play important roles in numerous biological processes such as tissue morphogenesis, differentiation and homeostasis. Furthermore, various diseases are caused by the dysfunction of ECM proteins. Therefore, identifying these important ECM proteins may assist in understanding related biological processes and drug development. In view of the serious imbalance in the training dataset, a Random Forest-based ensemble method with hybrid features is developed in this paper to identify ECM proteins. Hybrid features are employed by incorporating sequence composition, physicochemical properties, evolutionary and structural information. The Information Gain Ratio and Incremental Feature Selection (IGR-IFS) methods are adopted to select the optimal features. Finally, the resulting predictor termed IECMP (Identify ECM Proteins) achieves an balanced accuracy of 86.4% using the 10-fold cross-validation on the training dataset, which is much higher than results obtained by other methods (ECMPRED: 71.0%, ECMPP: 77.8%). Moreover, when tested on a common independent dataset, our method also achieves significantly improved performance over ECMPP and ECMPRED. These results indicate that IECMP is an effective method for ECM protein prediction, which has a more balanced prediction capability for positive and negative samples. It is anticipated that the proposed method will provide significant information to fully decipher the molecular mechanisms of ECM-related biological processes and discover candidate drug targets. For public access, we develop a user-friendly web server for ECM protein identification that is freely accessible at http://iecmp.weka.cc.

## Introduction

Extracellular matrix (ECM) is an important part of the cellular microenvironment and has a major regulatory or instructive influence on properties of tissues and cell behavior [1–4]. The ECM can sense and transduce signals that impact cell fate decisions [5]. The functions of ECM are reflected in the diversity of ECM proteins [6]. Previous study provided evidence that an ECM protein can facilitate specific tissue differentiation during embryonic development [7]. In addition, ECM proteins have an effect on the regulation of angiogenesis [8]. The protein composition and dynamics of the ECM are of crucial importance for numerous biological events such as tissue morphogenesis, differentiation and homeostasis [9].

Two main classes of ECM proteins are proteoglycans and collagens. Proteoglycans regulate a wide variety of biological activities, including tissue repair, tumor growth, cellular adhesion, proliferation, and migration [10]. Collagens are widely used in bone tissue engineering applications [1], provide tensile strength, regulate cell adhesion, support chemotaxis and migration, and direct tissue development [2]. Thus accurate identification of ECM proteins may provide important clues to decipher the underlying mechanisms in the above-mentioned biological processes and design ECM protein based biomaterials for bone tissue engineering applications.

As crucial modulators of cell behavior, ECM proteins have been implicated in numerous human diseases [4, 10]. The ECM protein, collagen VI, was found to be a crucial determinant of muscle disorders including severe congenital muscular dystrophy and milder Bethlem myopathy [11]. The decorin, a member of proteoglycan gene family, plays an important role in suppressing cancer cell growth and metastasis [12]. Therefore, ECM is a source of diagnostic and prognostic biomarkers [4]. To assist in patient diagnosis, an urgent need is to identify the related ECM proteins in development and pathology. The effort to identify ECM proteins may open novel opportunities for mechanistic understanding of disease pathogenesis as well as provide pathology-specific biochemical markers. What’s more, new identified proteins may provide key information for biomedical applications, including wound healing, tissue regeneration, and the rational design of mimetic biomaterials [3].

With the avalanche of genome sequences generated in the postgenomic age, it would be of great benefit to develop computational methods for rapidly and effectively identifying ECM proteins [13]. To our best knowledge, three machine-learning methods have been proposed in recent years to predict ECM proteins. Juan J et al. [14] presented a predictor called ECMPP (ECM Protein Prediction) which introduced five novel characteristics of ECM proteins, including molecular weight, sequence length, repetitive residue, repeated domains, and glycine-x-y repeats. Anitha J et al. [15] proposed a computational method (referred to in this paper as PECMP (Prediction of ECM Protein)) for prediction of extracellular matrix proteins using position specific scoring matrix as input features for the SVM^*hmm*^ Classifier. Kandaswamy KK et al. [16] provided a web server, ECMPRED (ECM PREDiction), to predict ECM proteins through a Random Forest approach based on features from the frequency of functional groups and physicochemical properties. The above methods have their own merits and achieve satisfactory results. However, two limitations should be noted. (i) The existing methods did not consider the sequence order and structure information which have been shown useful for protein attribute prediction [17]. (ii) Earlier work did not deal with the class imbalance problem. In this case, the classifier would tend to predict most of the incoming data belonging to the majority class, which limited the prediction performance.

**Fig 1:**
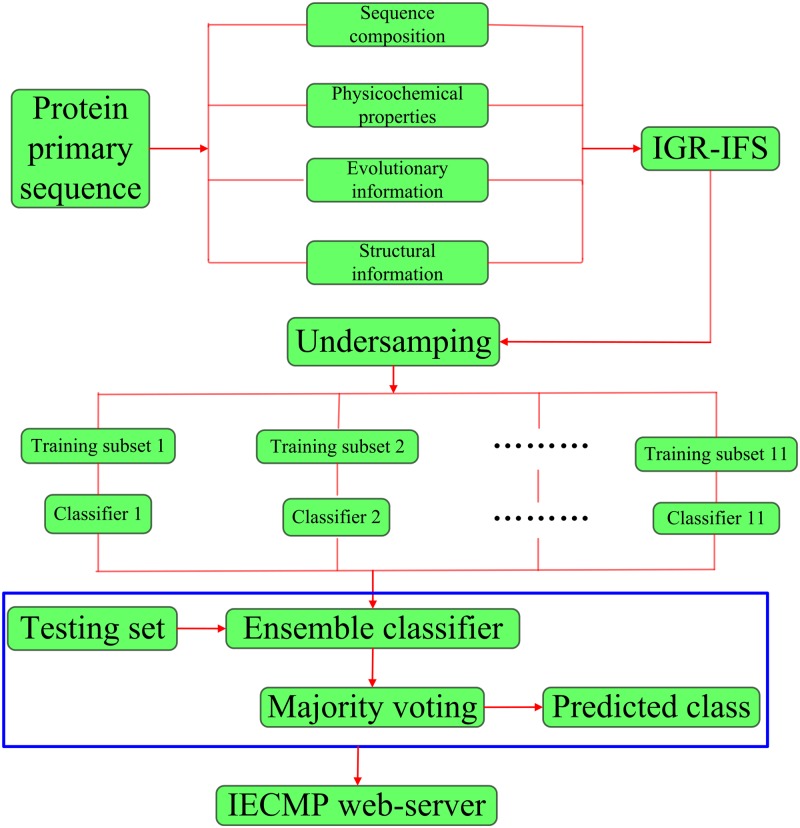
The overall work flow of the proposed method IECMP(Identify ECM Proteins). (i) The training sequences are mapped into feature vectors. (ii) To reduce the complexity and the feature redundancy, the Information Gain Ratio and Incremental Feature Selection (IGR-IFS) methods are employed. (iii) The training set is divided into 11 training subsets through the undersampling approach. (iv) With the optimal features, the 11 training subsets train Random Forest classifiers, respectively. (v) The predicted class labels of the test set are determined by the majority voting method.

To address these two limitations and enhance the prediction performance, we present an ensemble method based on hybrid features to identify ECM proteins. The proposed method, IECMP, is implemented in the following five steps. (i) The training sequences are mapped into feature vectors. To fully extract information from the original sequence, the process extracts hybrid features from sequence composition, physicochemical properties, evolutionary and structural information. (ii) To reduce the complexity and the feature redundancy, the Information Gain Ratio and Incremental Feature Selection (IGR-IFS) methods are employed. A training model is then built to determine a subset of the optimal features. (iii) The training set is divided into 11 training subsets through the undersampling approach. (iv) Based on the optimal features, the 11 training subsets train Random Forest classifiers, respectively. (v) The predicted class labels of the test set are determined through the majority voting method. The overall work flow of our method is shown in Fig. 1. For public easy to access and utilize, the presented approach is realized on a user-friendly IECMP web-server.

The average of sensitivity and specificity named balanced accuracy is presented to evaluate the performance, which is able to recover the drawback of accuracy regarding unbalanced data. The presented method IECMP achieves a higher balanced accuracy for the 10-fold cross validation and the test set. Our comparison results demonstrate that the proposed approach is superior to the existing methods on the balanced accuracy index for both the training dataset and the independent testing dataset.

## Materials and Methods

### 1. Datasets

In biology, the extracellular matrix (ECM) is a collection of extracellular molecules secreted by cells, such as structural proteins, matricellular proteins, and proteoglycans, which influences the mechanical properties of tissues and the phenotype of the cells that reside in those tissues [2]. The ECM proteins include both structural components of the ECM and matricellular elements. Furthermore, the ECM is refered to as a subcellular location in the UniProt Knowledgebase. In this paper, the ECM protein dataset is as well composed of proteins that locate in the ECM while the non-ECM protein dataset is composed of proteins that don’t locate in the ECM. The datasets used in this paper for performance analysis and comparison are divided into two parts: training dataset and independent testing dataset.

A dataset composed of 445 ECM proteins and 4486 non-ECM proteins, introduced by Kandaswamy KK et al. [16], is employed to construct the origin training dataset. The dataset is obtained from metazoan secreted protein sequences. The details about the procedure to obtain the dataset can be found in [16].

In order to compare with reported methods on dataset not used for training, an independent testing dataset, not including the training samples, is obtained from the human proteome. The human ECM proteins are extracted from the Supplemental File 3 of the reference [6], which includes 159 proteins. The human non-ECM proteins are collected from the dataset Hum3681 introduced by reference [18], which includes 3681 proteins. The human ECM proteins and non-ECM proteins are combined together to construct the original independent testing dataset. The final training dataset and independent testing dataset are obtained from the origin training dataset and independent testing dataset (original datasets) through the following two steps.

**Step 1**: To obtain high quality data, protein sequences of the origin datasets with less than 50 amino acids or more than 3000 amino acids are screened out. Protein sequences deleted from Unipro or containing ambiguous amino acids, such as B, J, O, U, X, and Z, are also removed from the origin datasets. To evaluate the proposed method objectively, protein sequences that are included in the training dataset are also screened out from the original independent testing dataset.

**Step 2**: After the first step, there are 3416 non-ECM protein sequences remaining in the ECM dataset and 85 ECM protein sequences remaining in the non-ECM dataset. However, in the feature extraction stage, it is time consuming for protein sequences to obtain the features extracted from secondary structure by PSIPRED. Aside from the ECM, the protein sequences in the Hum3681 locate in altogether 13 human subcellular locations. Without loss of generality, to reduce the computational complexity, we randomly select 10 sequences from each of the 13 subcellular locations in the remaining 3416 non-ECM protein sequences to form the non-ECM set. In addition, we performed an experiment to evaluate our method on the non-ECM dataset that randomly select 300 sequences from the Hum3681 dataset. Results show that the performance of this case is similar to that given in the paper. Therefore, it is reasonable and timesaving to evaluate our method by randomly selecting 10 sequences from each of the 13 subcellular locations.

Thus the final training dataset consists of 410 metazoan ECM proteins and 4464 metazoan non-ECM proteins while the final independent testing dataset consists of 85 human ECM proteins and 130 human non-ECM proteins. The final training and independent testing datasets are available in S1 Table.

### 2. Feature Extraction

To develop a computational method for predicting protein attributes, protein sequences should be represented as a feature vector that could really reflect the intrinsic correlation with the desired target [13]. Constructing a proper feature vector of proteins is a key step for a successful prediction [19]. As discussed in [20], an individual feature extraction strategy does not preserve enough discriminative information. The idea of hybrid model can be adopted for enhancing the discrimination power [21]. To realize this, protein sequences are characterized by the hybrid features based on sequence composition, physicochemical properties, evolutionary and structural information. Details about these descriptors are given in following parts.

#### 2.1. Feature Extraction based on Sequence Composition

**(I)Frequencies of functional groups.** It has been reported that side chains of amino acids perform a significant role in formatting and folding of proteins structure [22]. We categorize amino acids into 10 functional groups based on the presence of side chain chemical groups such as phenyl (F/W/Y), carboxyl (D/E), imidazole (H), primary amine (K), guanidino (R), thiol (C), sulfur (M), amido (Q/N), hydroxyl (S/T) and non-polar (A/G/I/L/V/P) [23]. The frequencies of the 10 functional groups are respectively computed for every sequence.

**(II)Information entropy.** The statistical distribution of amino acids contains uncertain information as a result of evolution. In information theory, entropy is a measure of the uncertainty [24]. The Shannon entropy, one of the most important metrics in information theory, is calculated using the following formula [25].

$$\begin{array}{c} H\left(x\right)=-\sum_{i=1}^{n}P_{i}\log_{2}\left(P_{i}\right). \end{array}$$ (1)

According to (1), we compute the Shannon entropy of amino acid composition and dipeptide composition, where *P*_*i*_(*i* = 1,2, ⋯,*n*) are the occurrence frequencies of 20 native amino acids or 400 dipeptides in protein sequences.

**(III)Distribution.** The count of each native amino acid in protein sequences is denoted by *N*_*i*_(*i* = 1,2, ⋯,20). $D_{j}^{i}$ is the distance from the *jth* amino acid *i* of the protein sequence to the first amino acid *i*. Then the distribution of amino acid *i* is

$$\begin{array}{c} D_{i}=\sum_{j=1}^{N_{i}}\frac{{\left(D_{j}^{i}-\frac{\sum_{j=1}^{N_{i}}D_{j}^{i}}{N_{i}}\right)}^{2}}{N_{i}}. \end{array}$$ (2)

**(IV)Transition.** To avoid losing order information hidden in protein sequences, the transition descriptor introduced by Dubchak I et al. [26] is applied to characterize the sequences. This feature set has been used for predicting membrane protein types [27], and DNA-binding proteins [28]. The transition descriptor is defined as

$$\begin{array}{c} T_{\alpha_{i}\alpha_{j}}=\frac{N_{\alpha_{i}\alpha_{j}}+N_{\alpha_{j}\alpha_{i}}}{L}, \end{array}$$ (3)

where *i*,*j* ∈ {1,2, ⋯,10} and *i* ≠ *j*. *α*_*i*_ is one of the above-mentioned 10 functional groups. *N*_*α*_*i*_*α*_*j*__ is the number of the dipeptide encoded as “*α*_*i*_ *α*_*j*_” in the sequence and *L* is the length of the sequence.

#### 2.2. Feature Extraction based on Physicochemical Properties

**(I)Pseudo amino acid composition.** The specificity and diversity of protein’s structure and function are largely related to various physicochemical properties of amino acids. The PseAAC (Pseudo Amino Acid Composition) method proposed by K.C. Chou can combine physicochemical properties with sequence order information properly [29], which has been widely applied in protein attribute prediction [13]. Meanwhile, various modes of PseAAC that extract different features from protein sequences were proposed in [30]. In this work, we adopt the PseAAC model presented in previous study [31]. Let the parameter *η* = 20, then 40 features are obtained from this model. The details about the model can be found in [31].

Four physicochemical properties including hydrophobicity, flexibility, net charge, and average accessible surface area are taken into account to calculate the model on the basis of the following reasons. (i) The hydrophobic effect is considered as the most important factor to affect protein structures [32]. (ii) The ECM protein collagen introduces flexibility into the molecules, which is crucial in regulating cell behavior [4]. (iii) Charged amino acids tend to form hydrogen bonds which are beneficial for ECM proteins in contact with solvents [33]. (iv) Previous studies have indicated that the accessible surface area of an amino acid is related to posttranslational modification [34], which may be the drive force for the ECM to form a dynamic network.

**(II)Discrete wavelet transform.** Discrete Wavelet Transform (DWT) can allow the analysis of signals both in time and frequency domain [35]. Thus DWT has achieved vast improvement in investigations of molecular biology data, such as genome sequence analysis [36], protein structure prediction [37], and gene expression data analysis [38]. DWT analysis can decompose the signals into the approximation coefficients, which represent the high-scale and low-frequency components of the signal, and the detail coefficients, which represent the low-scale and high-frequency components of the signal [39]. We apply DWT on the numerical signal converted from the three physicochemical properties, hydrophobicity, flexibility, and average accessible surface area, respectively.

The following statistical features are calculated for the identification of ECM proteins. (i) Mean, and standard deviation of the original signal. (ii) Maximum, minimum, mean, and standard deviation of the wavelet coefficients in each sub-band. The “Db4” wavelet function is selected and the decomposition level 4 is chosen. Finally, 42 features are obtained for every sample.

#### 2.3. Feature Extraction based on Evolutionary Information

Evolutionary conservation usually reflects important biological function [40]. Previous studies have proven that evolutionary information is important in protein structure and function predictions [41, 42]. To incorporate the evolutionary information of proteins, the position specific scoring matrix (PSSM) [43] profiles are adopted here. The PSSM is calculated by runnning PSI-Blast program through three iterations with 0.001 as the E-value cutoff. For a protein sequence with *L* residues, the generated PSSM matrix includes *L*×20 elements, which can be expressed as

$$\begin{array}{c} P_{\mathrm{PSSM}}=\left[\begin{array}{cccccc} E_{1\to 1} & E_{1\to 2} & \cdots & E_{1\to j} & \cdots & E_{1\to 20}\\ E_{2\to 1} & E_{2\to 2} & \cdots & E_{2\to j} & \cdots & E_{2\to 20}\\ \vdots & \vdots & \cdots & \vdots & \cdots & \vdots \\ E_{i\to 1} & E_{i\to 2} & \cdots & E_{i\to j} & \cdots & E_{i\to 20}\\ \vdots & \vdots & \cdots & \vdots & \cdots & \vdots \\ E_{L\to 1} & E_{L\to 2} & \cdots & E_{L\to j} & \cdots & E_{L\to 20} \end{array}\right], \end{array}$$ (4)

where *E*_*i* → *j*_ represents the score of the amino acid in the *ith* position of the query sequence being mutated to amino acid type *j* during the evolution process. The elements of PSSM are normalized using the following sigmoid function.

$$\begin{array}{c} f\left(x\right)=\frac{1}{1+e^{-x}}, \end{array}$$ (5)

where *x* is the original PSSM value. The protein *P* is represented as

$$\begin{array}{c} F_{\mathrm{PSSM}}=\left[\theta_{1}^{1},\theta_{2}^{1},\cdots ,\theta_{20}^{1},\theta_{1}^{2},\theta_{2}^{2},\cdots ,\theta_{20}^{2},\cdots ,\theta_{1}^{\lambda },\theta_{2}^{\lambda },\cdots ,\theta_{20}^{\lambda }\right], \end{array}$$ (6)

where $\theta_{i}^{\lambda }=\frac{1}{L-\lambda }\sum_{j=1}^{L-\lambda }{\left(E_{j\to i}-E_{j+\lambda \to i}\right)}^{2}$, *i* = 1, 2, ⋯ 20, 0 < λ < *L*. The value of *λ* is chosen as 4. Thus the total number of extracted features from evolutionary information equals 80.

#### 2.4. Feature Extraction based on Structural Information

**(I)Disorder.** A protein region is defined as “disorder” if it fails to fold into a stable three-dimensional structure. The disorder regions play important roles in various signaling and regulatory pathways such as transcriptional regulation, cellular signal transduction, and posttranslational modification [44]. The disorder predictor “VSL2” [45] is employed to calculate the disorder score of each residue in a given protein sequence. The following 8 features are designed to encode each protein sequence. (i) Mean/standard deviation of all residues’ disorder scores (2 features). (ii) Number of disorder/non-disorder segments (2 features). (iii) Minimum/maximum length of disorder/non-disorder segments (4 features).

**(II)Secondary structural information.** Previous studies have demonstrated that protein secondary structure results in the formation of tertiary structure, which reveals the function of proteins to a great extent [46, 47]. The secondary structure has 3 regular conformations, alpha helix (H), beta strand (E), and random coil (C). In this study, an effective tool PSIPRED [48] is employed to map protein sequences into secondary structural sequences. The following 51 features are computed from secondary structure information. (i) Number of helix/strand/coil divided by the sequence length (3 features). (ii) Number of the helix/strand/coil segments divided by the total number of secondary structure segments (3 features). (iii) Distribution of helix, strand, and coil (3 features). (iv) Minimum/maximum/mean/standard deviation of the length of the helix/strand/coil segments (12 features). (v) Frequencies of helix/strand/coil in 10 functional groups (30 features).

**(III)Functional domain information.** Protein domains can evolve, function, and exist independently and carry out diverse molecular functions [49]. Previous studies have demonstrated that proteins in the same organelle share specific functional domains [50, 51]. Furthermore, ECM proteins are typically made up of distinct domains for protein interactions. Therefore, we perform the feature extraction work from the functional domain information through the following steps. First, the functional domain composition of each ECM protein in the training dataset is obtained from the Intepro database [52]. Then, functional domains present in more than or equal to 25 ECM proteins are chosen to extract features. The result covers a total of 17 Intepro entries, which can be found in S2 Table. Finally, information of each of the 17 functional domains is represented by a binary score: 1 if present and 0 otherwise.

In this paper, each protein sample is encoded with hybrid features based on the above-mentioned sequence composition, physicochemical properties, evolutionary and structural information. Table 1 illustrates detailed information of the 315 features adopted in this study.

**Table 1:** Summary of the considered features. Hybrid features are employed by incorporating sequence composition, physicochemical properties, evolutionary and structural information.

Category	Feature type	No. of features
Sequence composition based (77 features)	Frequencies Of Functional Groups(FFG)	10
	Information entropy	2
	Distribution	20
	Transition	45
Physicochemical properties based (82 features)	Pseudo Amino Acid Composition(PseAAC)	40
	Discrete Wavelet Transformation(DWT)	42
Evolutionary information based(80 features)	Position Specific Scoring Matrix (PSSM)	80
Structural information based (76 features)	Disorder	8
	Secondary Structural Information(SSI)	51
	Functional Domain Information(FDI)	17

### 3. Feature Selection

After carrying out the feature extraction methods mentioned above, all protein sequences are converted into numerical feature vectors with the same dimension. However, the original feature set generally contains redundant or uninformative features which often result in poor prediction performance and dimension disaster [53]. To overcome these shortcomings, the Information Gain Ratio followed by Incremental Feature Selection (IGR-IFS) method is performed in current work to pick out informative features.

**Information Gain Ratio.** Information Gain Ratio (IGR) is a good measure of the relevance of an attribute with respect to classes [54]. In this paper, the entropy of the class *C* is defined as

$$\begin{array}{c} H\left(C\right)=-\sum_{j=1}^{2}P\left(C_{j}\right)\log_{2}P\left(C_{j}\right), \end{array}$$ (7)

where *P*(*C*_*j*_) is the percent of class *C*_*j*_ (ECM protein or non-ECM protein) in the training set.

The set of values of feature *F*_*i*_(*i* ∈ {1,2, ⋯,315}) is denoted as $S_{i}=\{V_{i}^{1},V_{i}^{2},\cdots ,V_{i}^{n_{i}}\}$. Then, the entropy of the feature *F*_*i*_ is expressed as

$$\begin{array}{c} H\left(F_{i}\right)=-\sum_{j=1}^{n_{i}}P\left(V_{i}^{j}\right)\log_{2}P\left(V_{i}^{j}\right). \end{array}$$ (8)

The conditional entropy of class *C*, given the feature *F*_*i*_, is defined as

$$\begin{array}{c} H\left(C\left|F_{i}\right.\right)=-\sum_{j=1}^{n_{i}}P\left(V_{i}^{j}\right)\sum_{k=1}^{2}P\left(C_{k}\left|V_{i}^{j}\right.\right)\log_{2}P\left(C_{k}\left|V_{i}^{j}\right.\right). \end{array}$$ (9)

The information gain ratio for the feature *F*_*i*_ is given by

$$\begin{array}{c} IGR\left(F_{i}\right)=\frac{H\;\left(C\right)-H\;\left(C\left|F_{i}\right.\right)}{H\;\left(F_{i}\right)}. \end{array}$$ (10)

According to this measure, *C* has a stronger correlation with *F*_*j*_ than with *F*_*i*_ if *IGR*(*F*_*j*_) > *IGR*(*F*_*i*_). The features then can be ranked by the IGR scores.

**Incremental Feature Selection.** Based on the ranked feature list evaluated by the IGR approach, the Incremental Feature Selection (IFS) method is adopted to determine the optimal feature set. The IFS procedure [55] starts with an empty feature set, and adds features one by one from higher to lower rank. A new feature set is constructed when another feature have been added. The feature set that has a relatively higher balanced accuracy and lower dimension is selected as the final input of the classification system.

### 4. Random Forest Classifier

The Random Forest (RF) algorithm, developed by L. Breiman [56], has excellent performance in protein attribute prediction problems [57, 58]. RF is an ensemble classifier consisting of several decision trees which are generated using a randomly sampled set of the original dataset. The predicted class is obtained by each of the constructed decision trees. The RF classifier then chooses the class with the most votes over all trees as the final prediction result. For detailed description about the RF algorithm, please refer to [56].

In this study, the Random Forest classifier in WEKA software [59] is employed to implement the classification with default parameters.

### 5. Ensemble Method

As described earlier in the **“Datasets”** section, the number of ECM proteins is much smaller than that of non-ECM proteins. This leads to the imbalanced data classification problem [60]. This issue will result in poor prediction accuracy of the minority class. The PECMP [15] and ECMPRED [16] have tried to change the distribution of positive and negative samples by randomly selecting ECM proteins and non-ECM proteins with a same size as the training set. However, they failed to make full use of the sample information in the original training dataset, which might adversely affect the prediction performance.

Previous studies have demonstrated that an ensemble classifier is often superior to the individual classifier, which enhances not only the performance of the classification, but also the confidence of the results [28, 61]. In this paper, the RF-based ensemble method is applied to address the imbalance problem. The prediction performance of the training dataset is evaluated by the 10-fold cross-validation as follows.

The positive and negative datasets are respectively divided into 10 subsets with an approximately equal number of samples. One subset from positive dataset and one subset from negative dataset are combined for testing, while the remaining subsets are used for training. The processes mentioned above are repeated 10 times. Each run, using the ensemble method, follows the two steps below.

**Step 1.** As the ratio of negative to positive samples is about 11, the negative dataset in the training set of each run is undersampled and split into 11 groups. Each group is then combined with the positive samples in the training set of each run as a training subset. After the undersampling procedure, 11 training subsets are obtained.

**Step 2.** 11 Random Forest classifiers are trained by the 11 training subsets, respectively and the performance of the model is evaluated by the testing set in each run. The final predicted class is determined by majority votes among the outputs of the 11 classifiers. In majority voting scheme, a test instance is labeled the predicted class that obtains the highest number of votes.

### 6. Performance Measures

In this study, 10-fold cross-validation and independent test are adopted to examine and compare the performance of ECM protein predictors. Sensitivity (*Sn*), specificity (*Sp*), accuracy (*Acc*), and balanced accuracy (*BAcc*) were employed to evaluate the performance of the prediction system. These measurements are defined as

$$\begin{array}{c} S_{n}=\frac{TP}{TP+FN}, \end{array}$$ (11)

$$\begin{array}{c} S_{p}=\frac{TN}{TN+FP}, \end{array}$$ (12)

$$\begin{array}{c} Acc=\frac{TP+TN}{TP+FP+TN+FN}, \end{array}$$ (13)

$$\begin{array}{c} BAcc=\frac{1}{2}\left(S_{n}+S_{p}\right), \end{array}$$ (14)

where *TP*, *TN*, *FP* and *FN* are the number of true positives, true negatives, false positives and false negatives, respectively.

Sensitivity and specificity reflect the rates of prediction accuracy with regard to positive and negative samples, respectively. Accuracy is the proportion of all samples that are correctly predicted. For the classification of unbalanced data, Accuracy is not an appropriate measure because it may be still high when the sensitivity is very low [62]. However, a good prediction system is usually expected to provide both high sensitivity and specificity. Therefore, the balanced accuracy is introduced as the main measure in this study.

## Results and Discussions

### 1. The Information Gain Ratio (IGR) Result

Based on the IGR algorithm mentioned in Section 2.3, the ranked feature list (see S3 Table) is obtained on the basis of each feature’s relevance to the class of samples. Within the list, a smaller index of a feature indicates that the feature is more important for ECM protein prediction. Such a list of ranked features are used to establish the optimal feature set in the IFS procedure.

### 2. IFS Result and the Optimal Feature Set

**Fig 2:**
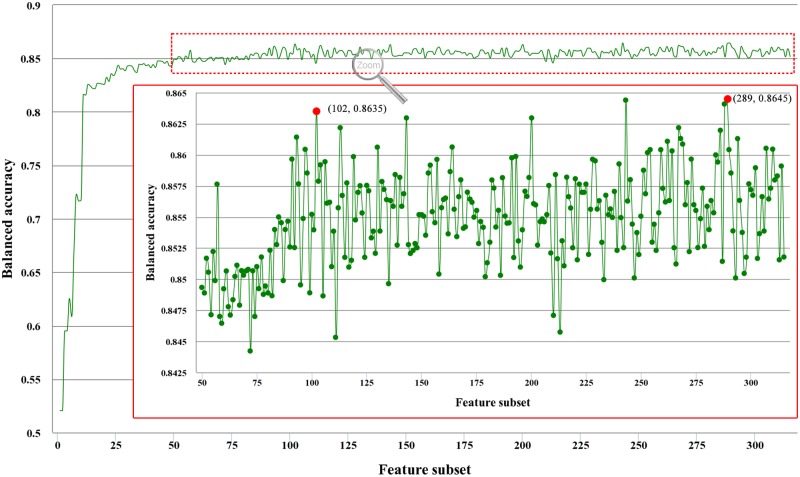
The IFS(Incremental Feature Selection) curve: the values of balanced accuracy against the feature subset. By adding features one by one from higher to lower rank, 315 different feature subsets are obtained. The individual predictor is then accordingly built for each feature subset and evaluated by 10-fold cross-validation. The IFS curve reveals the relation between the balanced accuracy (*BAcc*) and the feature subset.

By adding features one by one from higher to lower rank, 315 different feature subsets are obtained. The individual predictor is then accordingly built for each feature subset and evaluated by 10-fold cross-validation. The prediction performance for each of the 315 predictors (The IFS result) is given in S4 Table. The IFS curve is plotted in Fig. 2, which reveals the relation between the balanced accuracy and the feature subset. It can be observed that the maximal *BAcc* is 0.8645 when the feature set is comprised of 289 features. In addition, when the 102 features are included, the *BAcc* is 0.8635 as shown in Fig. 2. It is a drop of just 0.001 from the the maximal *BAcc*. To avoid dimension disaster, the 102 features (see S5 Table) are selected as the optimal feature set to identify ECM proteins.

To evaluate our feature selection method, Table 2 shows the prediction results of the original feature set and the optimal feature set. As can be seen from Table 2, the sensitivity, accuracy, and balanced accuracy of the optimal feature set are all superior to those of the original feature set. The specificity of the optimal feature set is also comparable to that of the original feature set. These results demonstrate that the original feature set truly contains redundant information or noise. The IGR-IFS method makes certain contribution to picking out informative features.

**Table 2:** Prediction results of the original feature set and the optimal feature set.

Feature set	No. of features	Sensitivity	Specificity	Accuracy	Balanced accuracy
Original feature set	315	0.854	0.850	0.850	0.852
Optimal feature set	102	0.878	0.849	0.851	0.864

### 3. Analysis of the Optimal Feature Set

**Fig 3:**
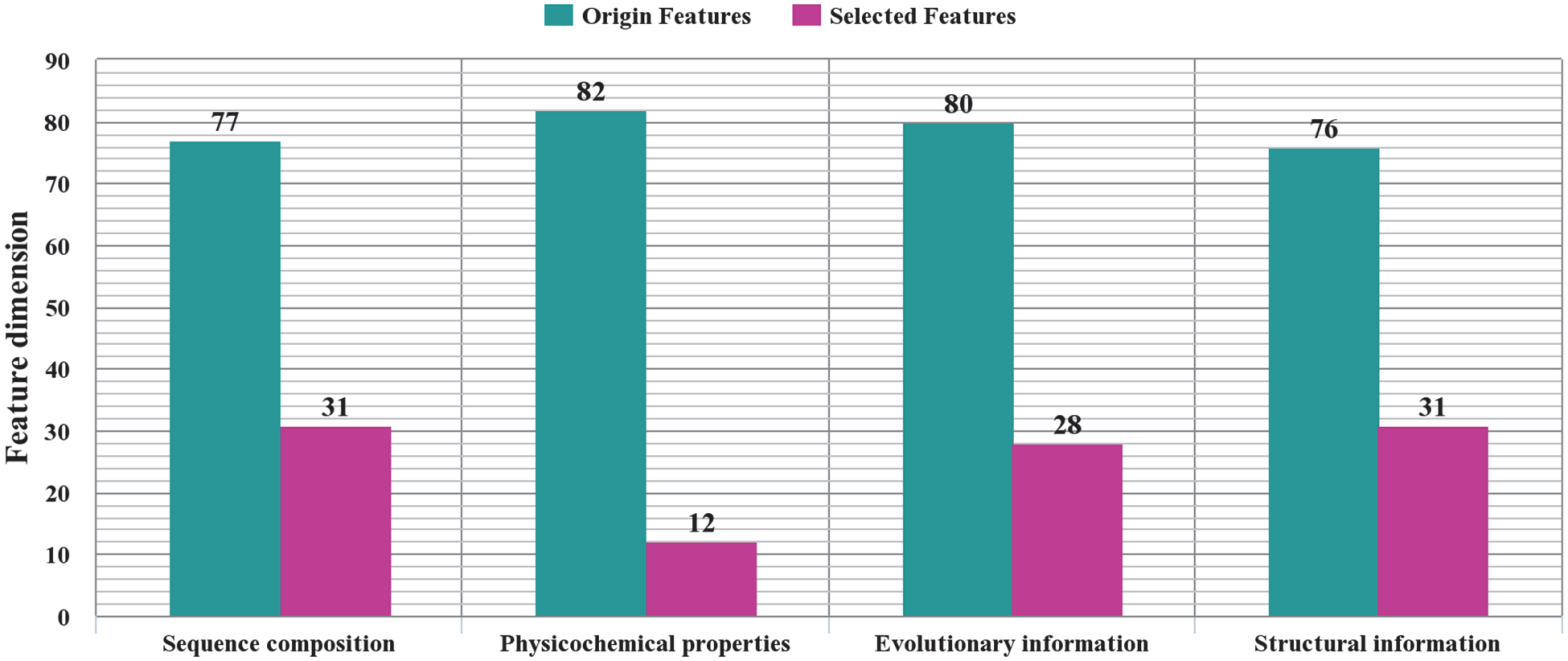
The numbers of each kind of features in the original and optimal feature set. The four kinds of features are based on sequence composition, physicochemical properties, evolutionary information, and structural information, respectively.

As described in “Feature Extraction” Section, there are four kinds of features derived from sequence composition, physicochemical properties, evolutionary and structural information. The numbers of each kind of features in the original and optimal feature set are depicted in Fig. 3. From the Figure, it can be seen that the numbers of the four kinds of features in the original feature set are rather close. After the feature selection, the sequence composition and structural information based features account for a high proportion of the optimal feature set (both are 31/102 = 0.304). This implies that the sequence composition and the structure of the protein are pivotal in determining the ECM proteins. However, the sequence order and structure information were largely ignored by past studies [14, 16]. Taking full advantage of the sequence order and structure information, this study is expected to improve the prediction performance.

**Fig 4:**
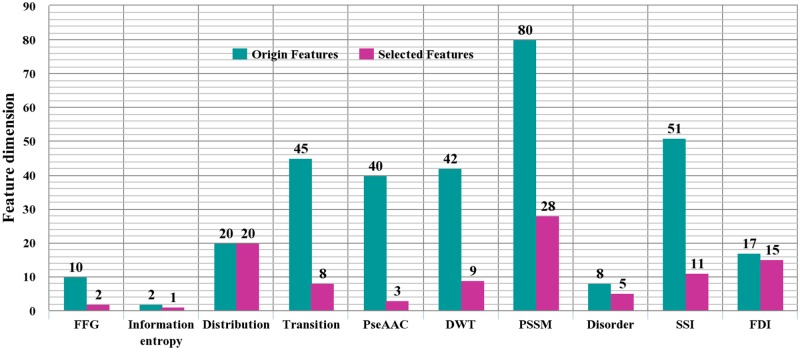
The feature type distributions in the original and optimal feature set. FFG: Frequencies of Functional Groups, PseAAC: Pseudo Amino Acid Composition, DWT: Discrete Wavelet Transformation, PSSM: Position Specific Scoring Matrix, SSI: Secondary Structural Information, FDI: Functional Domain Information.

The four kinds of features in Fig. 3 produce 10 types of feature vectors as given in Table 1. The feature type distributions in the original and optimal feature set are illustrated in Fig. 4. From Fig. 4, it is interesting to note that features from distribution are all in the optimal feature set. This phenomenon may be due to that the features from distribution reflect sequence order information, known to represent important properties for protein attribute prediction [23–25]. Furthermore, a much higher percent of features from functional domain information (15 out of 17) are selected from the original feature set. This finding is consistent with previous studies. In previous works, it was indicated that the key role of ECM in governing protein interactions is attributed to highly conserved domains of ECM proteins [63]. Although slightly less relevant, the other eight types of features also contribute to the identification of ECM proteins. The prediction model integrate multiple sources of descriptors for protein sequences in an attempt to enhance prediction performance.

### 4. Imbalanced Learning Effects

**Fig 5:**
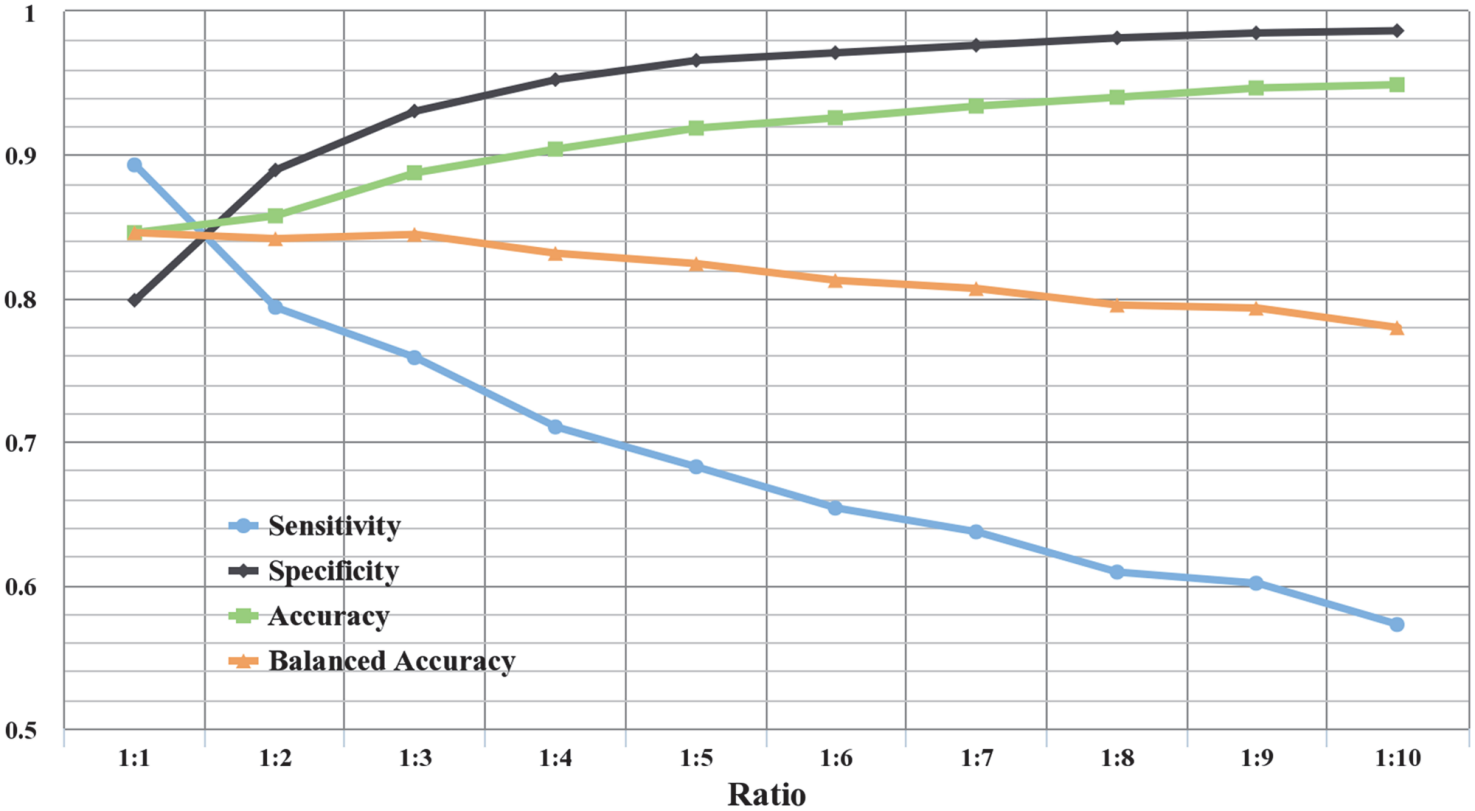
The averaged performance with different ratios between positive and negative samples in the training set. The ratios of the number of positive samples to negative ones are from 1:1 to 1:10, respectively.

To analyze the influence of the imbalanced problem on prediction performance, 10 training datasets are constructed by randomly extracting negative samples from the training dataset and plusing all positive samples in the training dataset. The ratios of the number of positive samples to negative ones in the 10 training datasets are from 1:1 to 1:10, respectively. Then the prediction results are evaluated on the 10 training datasets using 10-fold cross-validation. The processes mentioned above are repeated 10 times. The averaged performance of prediction systems trained with different positive to negative sample ratios is shown in Fig. 5 and listed in Table 3.

**Table 3:** The averaged performance with different ratios between positive and negative samples in the training set. The ratios of the number of positive samples to negative ones are from 1:1 to 1:10, respectively.

Ratio	Sensitivity	Specificity	Accuracy	Balanced accuracy
1:1	0.893	0.799	0.846	0.846
1:2	0.794	0.889	0.857	0.842
1:3	0.759	0.930	0.888	0.845
1:4	0.710	0.952	0.904	0.831
1:5	0.682	0.966	0.919	0.824
1:6	0.654	0.971	0.926	0.812
1:7	0.637	0.976	0.934	0.807
1:8	0.609	0.982	0.940	0.795
1:9	0.601	0.985	0.947	0.793
1:10	0.572	0.987	0.949	0.779

As shown in Fig. 5, the specificity is gradually improved with the increase of negative samples. On the contrary, the sensitivity keeps declining. This phenomenon demonstrates that the imbalanced problem will lead to most of samples classified as the majority class. These results also indicate that the prediction performance is indeed affected by the imbalanced training dataset. In addition, the accuracy has the opposite trend compared to the sensitivity, from 0.846 to 0.949, which indicates that the more serious the imbalance problem is, the higher accuracy it will be. Therefore, the accuracy is not a good measure for the imbalanced problem. But the balanced accuracy tends to drop accompanied with higher ratios (more imbalanced datasets). Therefore, for the imbalanced training dataset, it is reasonable that the balanced accuracy is chosen as the performance measure to select the optimal features.

In Table 3, a balanced training dataset with 1:1 ratio yields the best performance among different ratios, which has the highest sensitivity and balanced accuracy. The sensitivity, specificity, accuracy, and balanced accuracy are 0.893, 0.799, 0.846, and 0.846, respectively. Thus 1:1 is the suitable ratio of the training set to identify ECM proteins, which is in this study maintained through the predictor development process.

To evaluate the effectiveness of our ensemble method to overcome the imbalanced problem, Table 4 shows the prediction results with or without the ensemble method. In Table 4, without the ensemble method, the accuracy and specificity are 0.956 and 0.989. But the sensitivity is only 0.598 due to the imbalanced data size. On the other hand, the prediction performance achieves a balanced sensitivity (0.878) and specificity (0.849) with the ensemble method. These results suggest that the ensemble method could solve the imbalanced problem in the ECM protein training dataset.

**Table 4:** Prediction results with or without the ensemble method.

Method	Sensitivity	Specificity	Accuracy	Balanced accuracy
Without ensemble	0.598	0.989	0.956	0.793
With ensemble	0.878	0.849	0.851	0.864

### 5. Comparison with Several Machine Learning Methods

In this section, the prediction results of the RF classifier and other state-of-the-art classifiers are compared. The data mining software WEKA [59] including several machine learning techniques is employed to execute the comparisons. Their prediction results with the 10-fold cross-validation are listed in Table 5. From Table 5, the prediction results of all the 7 classifiers are quite close, which proves that our method is robust to classifiers. Specifically, the balanced accuracy of the RF classifier as recorded in Table 5 is 0.038, 0.057, 0.029, 0.013, and 0.019 higher than that of the Adaboost, BayesNet, Decisiontable, J48, and Logistic classifiers. Meanwhile, the performance of the RF is very close to that of the MLP, only 0.002 less for the balanced accuracy. However, the MLP is time-consuming during the classification process. These comparison results indicate that the RF is an ideal choice among different machine learning methods.

**Table 5:** Comparison with several machine learning methods.

Classifier	Sensitivity	Specificity	Accuracy	Balanced accuracy
Adaboost	0.868	0.784	0.791	0.826
BayesNet	0.824	0.791	0.793	0.807
Decisiontable	0.859	0.811	0.815	0.835
J48	0.871	0.832	0.835	0.851
Logistic	0.849	0.842	0.842	0.845
MLP	0.861	0.871	0.870	0.866
Random Forest	0.878	0.849	0.851	0.864

### 6. Comparison with the Existing Methods on the Training Dataset

To gain insights into the prediction power of our developed approach, we compare the prediction results of the IECMP approach with results from the existing approaches, ECMPP [14], PECMP [15], and ECMPRED [16], are compared. The method ECMPP [14] generates five novel features specific to ECM proteins for ECM protein prediction. The method PECMP [15] develops a SVM^*hmm*^ based model using PSSM profiles to facilitate the identification of ECM proteins. More recently, ECMPRED [16] employs an RF classifier trained on frequencies of functional groups and physicochemical properties for classifying ECM proteins. To make a fair comparison, these methods are trained on the same training dataset as introduced in Section 2.1. The prediction results of above-mentioned methods using the 10-fold cross-validation are summarized in Table 6.

**Table 6:** The prediction results compared with other methods on the training dataset using 10-fold cross-validation.

Reference	Method	Sensitivity	Specificity	Accuracy	Balanced accuracy
[14]	ECMPP	0.563	0.992	0.956	0.778
[15]	PECMP	0.490	0.971	0.931	0.731
[16]	ECMPRED	0.650	0.770	0.830	0.710
This study	IECMP	0.878	0.849	0.851	0.864

As shown in Table 6, it is obvious that PECMP achieves the lowest sensitivity (0.490). The method ECMPRED has the lowest accuracy (0.830) and specificity (0.770). On the contrary, ECMPP achieves the highest accuracy (0.956) and specificity (0.992). However, the sensitivity of ECMPP is extremely poor, 0.315 lower than that of IECMP. IECMP also achieves the highest balanced accuracy (0.864), followed by ECMPP with 0.778, PECMP with 0.731, and ECMPRED with 0.710. In other words, it can obtain better trade-off between sensitivity and specificity. Therefore, our method is superior to ECMPP, PECMP, and ECMPRED in regard to both high sensitivity and specificity.

### 7. Comparison with the Existing Methods on the Independent Testing Dataset

To further demonstrate the efficiency of the proposed model and avoid biased evaluations, it is objective to compare the performance of IECMP with those of previous methods on an independent dataset. Table 7 reports the detailed prediction results obtained by ECMPP, PECMP, ECMPRED, and IECMP on the independent dataset given in Section 2.1. To gain a fair comparison result, these methods are all trained with the same training dataset adopted in this study.

**Table 7:** The prediction results compared with other methods on the independent testing dataset.

Reference	Method	Sensitivity	Specificity	Accuracy	Balanced accuracy
[14]	ECMPP	0.294	0.985	0.712	0.640
[15]	PECMP	0.435	0.938	0.740	0.687
[16]	ECMPRED	0.622	0.478	0.535	0.550
This study	IECMP	0.765	0.785	0.777	0.775

In Table 7, ECMPP achieves the lowest sensitivity of 0.294 and the highest specificity of 0.985, which may be attributed to the imbalanced training dataset (410 positive samples and 4464 negative samples). PECMP also has a relatively low sensitivity (0.435) and a relatively high specificity (0.938). Although trained with an imbalanced training dataset (410 positive samples and 410 negative samples), ECMPRED obtains the lowest specificity of 0.478 and the lowest of balanced accuracy of 0.550. This phenomenon may be due to the fact that ECMPRED fails to make full use of the negative sample information in the original training dataset. Furthermore, for ECMPP, PECMP, and ECMPRED, there is a great divergence between sensitivity and specificity. In contrast, with a sensitivity of 0.765 and a specificity of 0.785, our method IECMP has a relatively balanced performance in positive and negative datasets. In terms of the balanced accuracy, the value of IECMP is 0.775, which is much better than ECMPP, PECMP, and ECMPRED. The outstanding performance of the current method may be attributed to the informativeness of the feature vector in representing proteins and the RF ensemble method. The good performance on the independent dataset also indicates that our method is robust to datasets.

### 8. Web-Server

**Fig 6:**
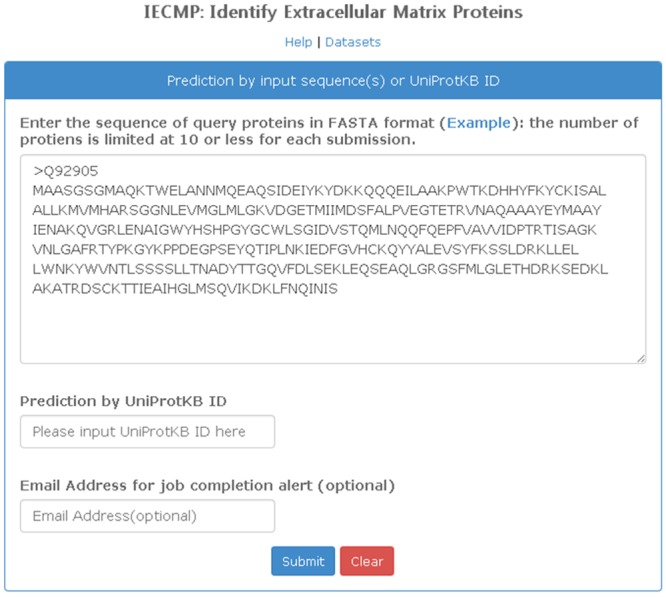
The main page of the IECMP web-server. Users can either enter the sequence of query proteins in FASTA format or input the UniProtKB ID of the query protein for prediction.

**Fig 7:**
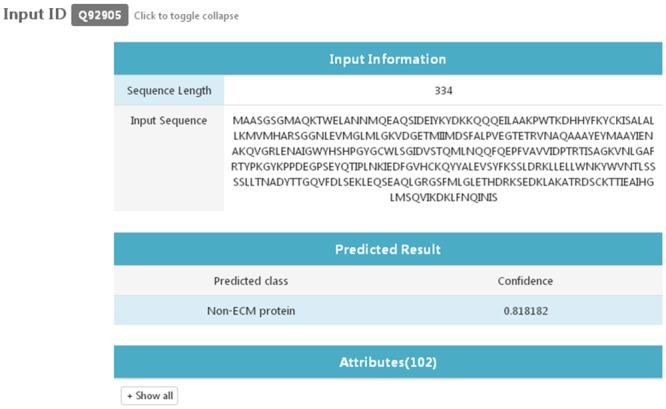
The predicted result page of the IECMP web-server. The predicted result page returns the input information, predicted result, and values of attributes for every submitted sequence.

To make it easy for public to access and utilize the method presented in this paper, an IECMP web-server has been launched and is freely available at http://iecmp.weka.cc. The main page of the IECMP web-server is shown in Fig. 6, while the predicted result page is shown in Fig. 7. As displayed in Fig. 6, users can either enter the sequence of query proteins in FASTA format or input the UniProtKB ID of the query protein for prediction. When protein sequences are submitted to the server, a job ID is presented to users. The predicted result page as shown in Fig. 7 will return the input information, predicted result, and values of attributes for every submitted sequence. If users enter your email address in the input box, predicted results will be emailed to users once the job has completed.

## Conclusions

Owing to the significance of ECM proteins in numerous biological events and human diseases, an urgent need is to develop a high-quality prediction model for identifying ECM proteins. An RF based ensemble method for ECM protein prediction is presented with hybrid features incorporating sequence composition, physicochemical properties, evolutionary and structural information. To enhance prediction performance, the IGR-IFS method is employed to select highly discriminating features. Experimental results show that our method IECMP obtains satisfactory results. The sensitivity, specificity, and balanced accuracy are 0.878, 0.849 and 0.851, respectively for the training set using 10-fold cross validation. When performed on the independent dataset, IECMP achieves a sensitivity of 0.765, a specificity of 0.785, and a balanced accuracy of 0.775. These results demonstrate that IECMP has a more balanced prediction capability for both positive and negative samples. Compared with prior studies, the proposed method not only took full advantage of multiple descriptors for protein sequences but also overcome the imbalanced data classification problem. The comparison results indicate that IECMP indeed performs better than the previous studies in terms of the balanced accuracy for both 10-fold cross-validation and independent test. It is anticipated that IECMP can provide candidate ECM proteins for future experimental verification to assist in understanding the molecular mechanisms of ECM-related biological processes and drug development for the treatment of human diseases.

## Supporting Information

S1 Table The training dataset and the independent testing dataset (.xlsx). The training dataset consists of 410 metazoan ECM proteins and 4464 metazoan non-ECM proteins while the independent testing dataset consists of 85 human ECM proteins and 130 human non-ECM proteins.
(XLSX)

S2 Table The 17 Intepro entries obtained from the Intepro database (.xlsx). Functional domains present in more than or equal to 25 ECM proteins are chosen to extract features. The result covers a total of 17 Intepro entries.
(XLSX)

S3 Table The ranked feature list given by the Information Gain Ratio algorithm (.xlsx). Within the list, a smaller index of a feature indicates that it is more important for ECM protein prediction. Such a list of ranked features were used to establish the optimal feature set in the IFS procedure.
(XLSX)

S4 Table The Incremental Feature Selection (IFS) result (.xlsx). By adding features one by one from higher to lower rank, 315 different feature subsets are obtained. The individual predictor is then accordingly built for each feature subset and evaluated by 10-fold cross-validation.
(XLSX)

S5 Table The optimal feature set (.xlsx). The maximal *BAcc* is 0.8645 when the feature set is comprised of 289 features. In addition, when 102 features are included, the *BAcc* is 0.8635, a drop of just 0.001 from the the maximal *BAcc*. To avoid dimension disaster, the 102 features are selected as the optimal feature set to identify ECM proteins.
(XLSX)

## References

[1] Mathews S, Bhonde R, Gupta PK, Totey S. (2012) Extracellular matrix protein mediated regulation of the osteoblast differentiation of bone marrow derived human mesenchymal stem cells. Differentiation 84: 185–192. 10.1016/j.diff.2012.05.001 22664173

[2] Rutnam ZJ, Wight TN, Yang BB. (2013) miRNAs regulate expression and function of extracellular matrix molecules. Matrix Biology 32: 74–85. 10.1016/j.matbio.2012.11.003 23159731 PMC4106267

[3] Muiznieks LD, Keeley FW. (2013) Molecular assembly and mechanical properties of the extracellular matrix: a fibrous protein perspective. Biochimica et Biophysica Acta 1832: 866–875. 10.1016/j.bbadis.2012.11.022 23220448

[4] Hubmacher D, Apte SS. (2013) The biology of the extracellular matrix: novel insights. Curr Opin Rheumatol 25: 65–70. 10.1097/BOR.0b013e32835b137b 23143224 PMC3560377

[5] Zhang J, Klos M, Wilson GF, Herman AM, Lian X, et al (2012) Extracellular matrix promotes highly efficient cardiac differentiation of human pluripotent stem cells: the matrix sandwich method. Journal of the American Heart Association 111: 1125–1136. 10.1161/CIRCRESAHA.112.273144 PMC3482164 22912385

[6] Cromar GL, Xiong X, Chautard E, Ricard-Blum S, Parkinson J. (2012) Toward a systems level view of the ECM and related proteins: a framework for the systematic definition and analysis of biological systems. Proteins 80: 1522–1544. 10.1002/prot.24169 22275077

[7] Endo Y, Ishiwata-Endo H, Yamada KM. (2012) Extracellular matrix protein anosmin promotes neural crest formation and regulates FGF, BMP, and WNT activities. Developmental Cell 23: 305–316. 10.1016/j.devcel.2012.07.006 22898776 PMC3422507

[8] Campbell NE, Kellenberger L, Greenaway J, Moorehead RA, Linnerth-Petrik NM, et al (2010) Extracellular matrix proteins and tumor angiogenesis. Journal of Oncology Article ID 586905. 10.1155/2010/586905 PMC2910498 20671917

[9] Chagnot C, Listrat A, Astruc T, Desvaux M. (2012) Bacterial adhesion to animal tissues: protein determinants for recognition of extracellular matrix components. Cellular Microbiology 14: 1687–1696. 10.1111/cmi.12002 22882798

[10] Karsdal MA, Nielsen MJ, Sand JM, Henriksen K, Genovese F, et al (2010) Extracellular matrix remodeling: the common denominator in connective tissue diseases. ASSAY and Drug Development Technologies 11: 70–92. 10.1089/adt.2012.474 PMC3593693 23046407

[11] Lampe AK, Bushby KM. (2005) Collagen VI related muscle disorders. J Med Genet 42: 673–685. 10.1136/jmg.2002.002311 16141002 PMC1736127

[12] Bi X, Tong C, Dockendorff A, Bancroft L, Gallagher L, et al (2008) Genetic deficiency of decorin causes intestinal tumor formation through disruption of intestinal cell maturation. Carcinogenesis 29: 1435–1440. 10.1093/carcin/bgn141 18550571 PMC2811538

[13] Chou KC. (2011) Some remarks on protein attribute prediction and pseudo amino acid composition. Journal of Theoretical Biology 273: 236–247. 10.1016/j.jtbi.2010.12.024 21168420 PMC7125570

[14] Jung J, Ryu T, Hwang Y, Lee E, Lee D. (2010) Prediction of extracellular matrix proteins based on distinctive sequence and domain characteristics. J Comput Biol 17: 97–105. 10.1089/cmb.2008.0236 20078400

[15] Anitha J, Rejimoan R, Sivakumar KC, Sathish M. (2012) Prediction of extracellular matrix proteins using SVMhmm classifier. IJCA Special Issue on Advanced Computing and Communication Technologies for HPC Applications 1: 7–11.

[16] Kandaswamy KK, Pugalenthi G, Kalies KU, Hartmann E, Martinetz T. (2013) EcmPred: prediction of extracellular matrix proteins based on random forest with maximum relevance minimum redundancy feature selection. Journal of Theoretical Biology 317: 377–383. 10.1016/j.jtbi.2012.10.015 23123454

[17] Liu B, Xu J, Zou Q, Xu R, Wang X, et al (2014) Using distances between Top-n-gram and residue pairs for protein remote homology detection. BMC Bioinformatics 15: Suppl 2:S3 10.1186/1471-2105-15-S16-S3 PMC4015815 24564580

[18] Li L, Zhang Y, Zou L, Li C, Yu B, et al (2012) An ensemble classifier for eukaryotic protein subcellular location prediction using gene ontology categories and amino acid hydrophobicity. PLoS ONE 7: e31057 10.1371/journal.pone.0031057 22303481 PMC3268814

[19] Bakhtiarizadeh MR, Moradi-Shahrbabak M, Ebrahimi M, Ebrahimie E. (2014) Neural network and SVM classifiers accurately predict lipid binding proteins, irrespective of sequence homology. Journal of Theoretical Biology 356: 213–222. 10.1016/j.jtbi.2014.04.040 24819464

[20] Hayat M, Tahir M, Khan SA. (2014) Prediction of protein structure classes using hybrid space of multi-profile Bayes and bi-gram probability feature spaces. Journal of Theoretical Biology 346: 8–15. 10.1016/j.jtbi.2013.12.015 24384128

[21] Zuo YC, Peng Y, Liu L, Chen W, Yang L, et al (2014) Predicting peroxidase subcellular location by hybridizing different 4 descriptors of Chou’s pseudo amino acid patterns. Analytical Biochemistry 458: 14–19. 10.1016/j.ab.2014.04.032 24802134

[22] Hayat M, Khan A. (2013) WRF-TMH: predicting transmembrane helix by fusing composition index and physicochemical properties of amino acids. Amino Acids 44: 1317–1328. 10.1007/s00726-013-1466-4 23494269

[23] Pugalenthi G, Kumar KK, Suganthan PN, Gangal R. (2008) Identification of catalytic residues from protein structure using support vector machine with sequence and structural features. Biochem. Biophys. Res. Commun 367: 630–634. 10.1016/j.bbrc.2008.01.038 18206645

[24] Robert MG. (2009) Entropy and Information Theory. Springer-Verlag New York Inc.

[25] Shannon CE. (1948) A mathematical theory of communication. Bell System Technical Journal 27: 379–423. 10.1002/j.1538-7305.1948.tb00917.x

[26] Dubchak I, Muchnik I, Holbrook SR, Kim SH. (1995) Prediction of protein folding class using global description of amino acid sequence. Proc Natl Acad Sci USA 92: 8700–8704. 10.1073/pnas.92.19.8700 7568000 PMC41034

[27] Han GS, Yu ZG, Anh V. (2014) A two-stage SVM method to predict membrane protein types by incorporating amino acid classifications and physicochemical properties into a general form of Chou’s PseAAC. Journal of Theoretical Biology 344: 31–39. 10.1016/j.jtbi.2013.11.017 24316387

[28] Zou C, Gong J, Li H. (2013) An improved sequence based prediction protocol for DNA-binding proteins using SVM and comprehensive feature analysis. BMC Bioinformatics 14: 90 10.1186/1471-2105-14-90 23497329 PMC3602657

[29] Chou KC. (2001) Prediction of protein cellular attributes using pseudo amino acid composition. Proteins: Structure, Function, and Bioinformatics 43: 246–255. 10.1002/prot.1035 11288174

[30] Shen HB, Chou KC. (2008) PseAAC: a flexible web-server for generating various kinds of protein pseudo amino acid composition. Anal. Biochem 373: 386–388. 10.1016/j.ab.2007.10.012 17976365

[31] Afridi TH, Khan A, Lee YS. (2012) Mito-GSAAC: mitochondria prediction using genetic ensemble classifier and split amino acid composition. Amino Acids 42: 1443–1454. 10.1007/s00726-011-0888-0 21445589

[32] Parisien M, Major F. (2007) Ranking the factors that contribute to protein beta-sheet folding. Amino Acids 65: 824–829. 10.1002/prot.21475 17523189

[33] Hayat M, Khan A. (2012) Mem-PHybrid: hybrid features based prediction system for classifying membrane protein types. Anal Biochem 424: 35–44. 10.1016/j.ab.2012.02.007 22342883

[34] Ahmad S, Gromiha MM, Sarai A. (2003) Real value prediction of solvent accessibility from amino acid. Proteins 50: 629–635. 10.1002/prot.10328 12577269

[35] Xiaoquan L, Hongde L, Zhonghua X, Qiang Z. (2004) Maximum spectrum of continuous wavelet transform and its application in resolving an overlapped signal. J. Chem. Inf. Comput. Sci 44: 1228–1237. 10.1021/ci0342977 15272830

[36] Vannucci M, Lio P. (2001) Non-decimated wavelet analysis of biological sequences: applications to protein structure and genomics. Sankhya B 63: 218–233.

[37] Sun XY, Shi SP, Qiu JD, Suo SB, Huang SY, et al (2012) Identifying protein quaternary structural attributes by incorporating physicochemical properties into the general form of Chou’s PseAAC via discrete wavelet transform. Molecular Biosystems 8: 3178–3184. 10.1039/c2mb25280e 22990717

[38] Myasnikova E, Samsonova A, Kozlov K, Samsonova M, Reinitz J. (2001) Registration of the expression patterns of Drosophila segmentation genes by two independent methods. Bioinformatics 17: 3–12. 10.1093/bioinformatics/17.1.3 11222257

[39] Mallat SG. (1989) A theory for multiresolution signal decomposition: the wavelet representation. IEEE Trans Pattern Anal Mach Intell 11: 674–693. 10.1109/34.192463

[40] Zuo YC, Peng Y, Liu L, Chen W, Yang L, et al (2014) Predicting peroxidase subcellular location by hybridizing different 4 descriptors of Chou’s pseudo amino acid patterns. Analytical Biochemistry 458: 14–19. 10.1016/j.ab.2014.04.032 24802134

[41] Ding S, Yan S, Qi S, Li Y, Yao Y. (2014) A protein structural classes prediction method based on PSI-BLAST profile. Journal of Theoretical Biology 353: 19–23. 10.1016/j.jtbi.2014.02.034 24607742

[42] Yang X, Guo Y, Luo J, Pu X, Li M. (2013) Effective Identification of Gram-Negative Bacterial Type III Secreted Effectors Using Position-Specific Residue Conservation Profiles. PLoS ONE 8: e84439 10.1371/journal.pone.0084439 24391954 PMC3877298

[43] Schaffer AA, Aravind L, Madden TL, Shavirin S, Spouge JL, et al (2001) Improving the accuracy of PSI-BLAST protein database searches with composition-based statistics and other refinements. Nucleic Acids Res 29: 2994–3005. 10.1093/nar/29.14.2994 11452024 PMC55814

[44] Dyson HJ, Wright PE. (2005) Intrinsically unstructured proteins and their functions. Nat. Rev. Mol. Cell Biol 6: 197–208. 10.1038/nrm1589 15738986

[45] Peng K, Radivojac P, Vucetic S, Dunker AK, Obradovic Z. (2006) Length dependent prediction of protein intrinsic disorder. BMC Bioinformatics 7: 208 10.1186/1471-2105-7-208 16618368 PMC1479845

[46] Landreh M, Astorga-Wells J, Johansson J, Bergman T, Jornvall H. (2011) New developments in protein structure-function analysis by MS and use of hydrogenCdeuterium exchange microfluidics. FEBS J 278: 3815–3821. 10.1111/j.1742-4658.2011.08215.x 21668648

[47] Qu W, Yang BR, Jiang W, Wang LJ. (2012) HYBP-PSSP: a hybrid back propagation method for predicting protein secondary structure. Neural Comput & Applic 21: 337–349. 10.1007/s00521-011-0739-7

[48] McGuffin LJ, Bryson K, Jones DT. (2000) The PSIPRED protein structure prediction server. Bioinformatics. 16: 404–405. 10.1093/bioinformatics/16.4.404 10869041

[49] Ashburner M, Ball CA, Blake JA, Botstein D, Butler H, et al (2000) Gene ontology: tool for the unification of biology. Nat. Genet 25: 25–29. 10.1038/75556 10802651 PMC3037419

[50] Hoglund A, Donnes P, Adolph HW, Kohlbacher O. (2005) From prediction of subcellular localization to functional classification: discrimination of DNA-packing and other nuclear proteins. Online J. Bioinform 6: 51–64.

[51] Chou KC, Cai YD. (2004) Prediction of protein subcellular locations by GO-FunD-PseAA predictor. Biochem. Biophys. Res. Commun 320: 1236–1239. 10.1016/j.bbrc.2004.06.073 15249222

[52] Mulder NJ, Apweiler R, Attwood TK, Bairoch A, Bateman A, et al (2000) InterPro—an integrated documentation resource for protein families, domains and functional sites. Bioinformatics 16: 1145–1150. 10.1093/bioinformatics/16.12.1145 11159333

[53] Saeys Y, Inza I, Larranaga P. (2007) A review of feature selection techniques in bioinformatics. Bioinformatics 23: 2507–2517. 10.1093/bioinformatics/btm344 17720704

[54] Yu L, Liu, H. (2003) Feature selection for high-dimensional data: a fast correlation-based filter solution. Proceedings of the Twentieth International Conference on Machine Learning.

[55] in H, Chen W, Ding H. (2013) AcalPred: a sequence-based tool for discriminating between acidic and alkaline enzymes. PLoS ONE 8: e75726 10.1371/journal.pone.0075726 24130738 PMC3794003

[56] Breiman L. (2001) Random forests. Machine Learning 45: 5–32. 10.1023/A:1010933404324

[57] Kandaswamy KK, Pugalenthi G, Hartmann E, Kalies KU, Moller S, et al (2010) SPRED: A machine learning approach for the identification of classical and non-classical secretory proteins in mammalian genomes. Biochemical and Biophysical Research Communications 391: 1306–1311. 10.1016/j.bbrc.2009.12.019 19995554

[58] Mohamed TP, Carbonell JG, Ganapathiraju MK. (2010) Active learning for human protein-protein interaction prediction. BMC Bioinformatics 11: Suppl 1:S57 10.1186/1471-2105-11-S1-S57 20122232 PMC3009530

[59] Witten IH, Frank E. (2005) Data mining: practical machine learning tools and techniques. San Francisco: Morgan Kaufmann.

[60] Asadabadi EB, Abdolmaleki P. (2013) Predictions of protein-protein interfaces within membrane protein complexes. Avicenna J Med Biotechnol 5: 148–157. 23919118 PMC3732864

[61] Hosseinzadeh F, Kayvanjoo AH, Ebrahimi M, Goliaei B. (2013) Prediction of lung tumor types based on protein attributes by machine learning algorithms. Springerplus 2: 238 10.1186/2193-1801-2-238 23888262 PMC3710575

[62] Weiss G. (2004) Mining with rarity: a unifying framework. SIGKDD Explorations 6: 7–19. 10.1145/1007730.1007734

[63] Hynes RO. (2009) The extracellular matrix: not just pretty fibrils. Science 326: 1216–1219. 10.1126/science.1176009 19965464 PMC3536535

